# Prospective Study about the Utility of Endoscopic Ultrasound for Predicting the Safety of Endoscopic Submucosal Dissection in Early Gastric Cancer (T-HOPE 0801)

**DOI:** 10.1155/2013/329385

**Published:** 2013-03-28

**Authors:** Daisuke Kikuchi, Toshiro Iizuka, Shu Hoteya, Akihiro Yamada, Tsukasa Furuhata, Satoshi Yamashita, Kaoru Domon, Masanori Nakamura, Akira Matsui, Toshifumi Mitani, Osamu Ogawa, Mitsuru Kaise

**Affiliations:** Department of Gastroenterology, Toranomon Hospital, 2-2-2 Toranomon, Minato-ku, Tokyo 105-8470, Japan

## Abstract

*Background*. Intraoperative bleeding is an important determinant for safety of endoscopic submucosal dissection (ESD) for early gastric cancer (EGC). This study aimed to prospectively evaluate the usefulness of endoscopic ultrasound (EUS) for predicting ESD safety. *Methods*. A total of 110 patients with EGC were divided into two groups based on EUS findings: group P, almost no blood vessels in submucosa, or ≤4 small vessels per field of view; group R, remaining patients. Primary endpoint was the decrease in Hb after ESD. Secondary endpoints included procedure time and the incidence of muscle injury and clip use. *Results*. A total of 89 patients were evaluated. Fifty were classified into group P and 39 into group R. Mean decrease in Hb was 0.27 g/dL in group P and 0.35 g/dL in group R, with no significant difference. Mean procedure time was significantly longer in group R (105.4 min) than in group P (65.5 min) (*P* < 0.001). The incidence of muscle injury and clip use were significantly higher in group R (25.6%/48.7%) than in group P (8.0%/20.0%) (*P* = 0.02/*P* = 0.004). *Conclusion*. Preoperative EUS can predict procedure time and the incidence of muscle injury and clip use and is thus considered useful for predicting gastric ESD safety.

## 1. Introduction

The advance from endoscopic mucosal resection (EMR) to endoscopic submucosal dissection (ESD) has enabled *en bloc* endoscopic resection of lesions, regardless of their location and size or the presence or absence of scar formation [1–3]. While ESD is now widely used for endoscopic treatment of early gastric cancer, there are several unsolved problems associated with its use, such as prolonged procedure time and high incidence of perforation, bleeding, and other complications [4]. In particular, intraoperative bleeding during gastric ESD significantly affects the safety of the procedure and can result in extra time for hemostasis and injury or perforation of the muscle layer due to loss of clear surgical field of view.

We have previously reported the use of endoscopic ultrasonography (EUS) to identify vasculature in the submucosa beneath the lesion and thereby predict the risk of intraoperative bleeding during ESD [5]. In this paper, we present the results of our prospective clinical study on the usefulness of EUS for predicting the intraoperative bleeding and safety of gastric ESD (Toranomon Hospital Prospective Study of Endoscopy: T-HOPE 0801). 

## 2. Materials and Methods

### 2.1. Patients

This study was commenced after obtaining approval from the Ethics Committee at Toranomon Hospital in June 2008. Inclusion criteria were (1) age ≥20 years, (2) gastric tumor treated by ESD, (3) having received EUS prior to ESD, and (4) having provided written informed consent. Patients were excluded if they (1) had multiple resectable gastric lesions, (2) had undergone any surgical procedure involving the stomach or esophagus, (3) had a lesion that extended to the esophagogastric junction or pyloric ring, (4) had a ulcer scar that made it difficult to identify the layer structure of the gastric wall by EUS, (5) had serious liver/kidney dysfunction or hematological disorder, (6) were receiving antiplatelet or anticoagulation therapy, or (7) were considered inappropriate for participation in this study by the attending physician.

**Figure 1:**
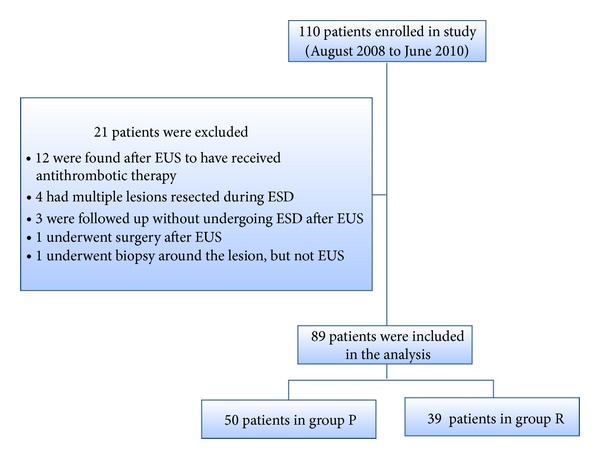
Flow of patients through this study.

A total of 110 patients were enrolled in this study between August 2008 and June 2010. Of them, 89 were included for analysis, and the following were excluded: 12 who were found after entry to be receiving antithrombotic therapy, 4 in whom multiple lesions were resected during ESD, 3 who were followed up without treatment after EUS, 1 who underwent surgery after EUS, and 1 who underwent stepwise biopsy, but not EUS, due to an irregularly bordered lesion (Figure 1).

### 2.2. EUS

**Figure 2:**
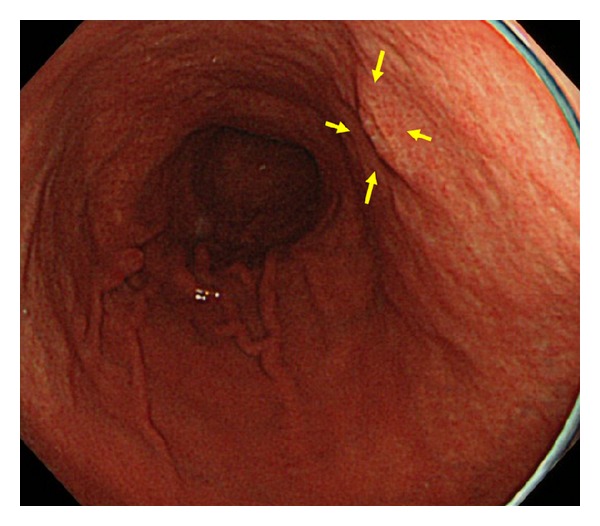
Endoscopic image of a patient in group P showing a flat elevated lesion on the posterior wall of the middle gastric body (yellow arrow).

**Figure 3:**
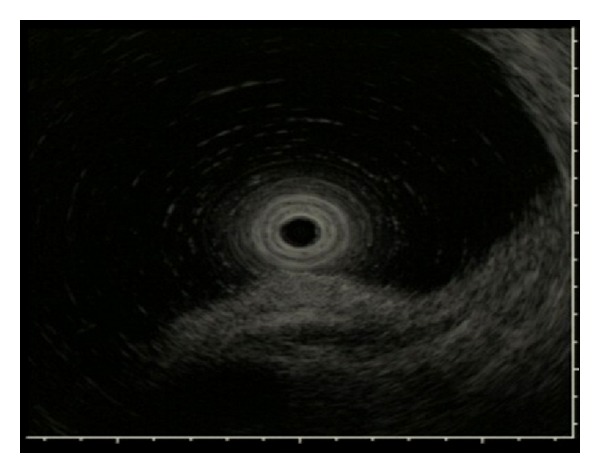
EUS image of the lesion of Figure 2. EUS showing no hypoechoic area suggestive of blood vessels in the third layer.

**Figure 4:**
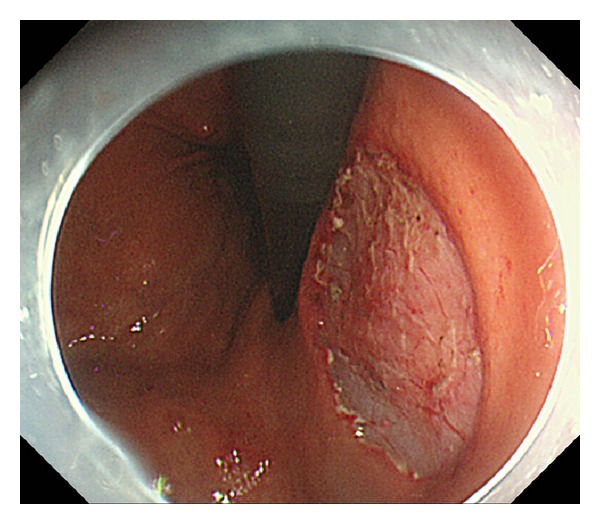
Post-ESD image of the lesion of Figure 2. There is no carbide on ESD ulcer.

**Figure 5:**
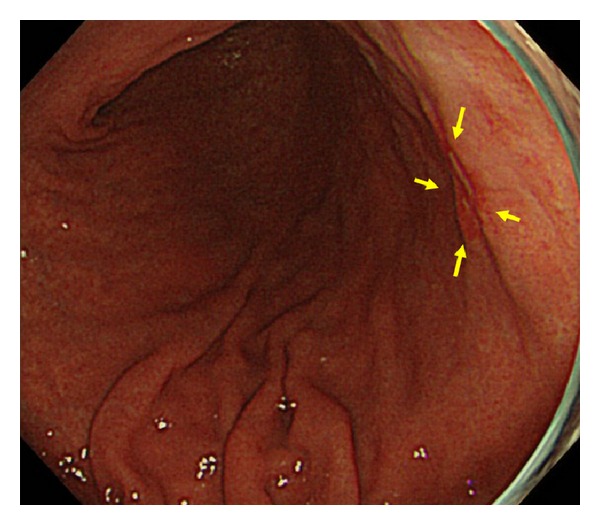
Endoscopic image of a patient in group R showing a depressed lesion on the posterior wall of the lower gastric body (yellow arrow).

**Figure 6:**
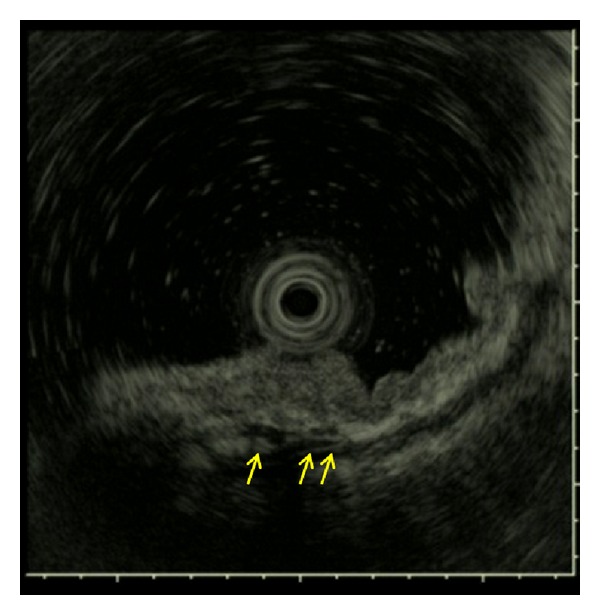
EUS image of the lesion of Figure 5. EUS showing some large vessels approximately 1000 *μ*m in diameter in the third layer (yellow arrow).

**Figure 7:**
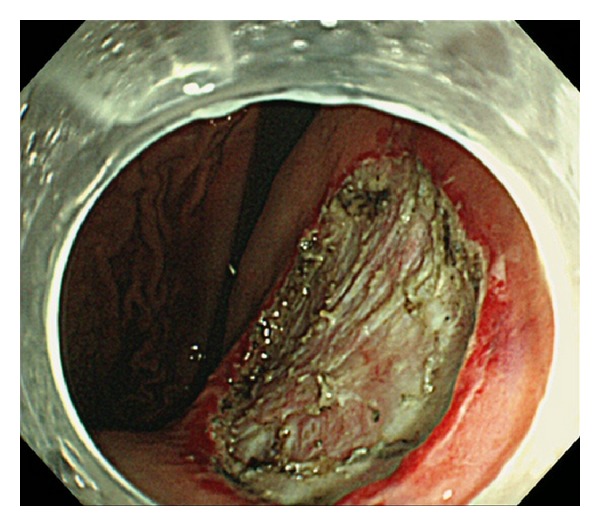
Post-ESD image of the lesion of Figure 5. A lot of carbide by hemostatic forceps and muscle injury were recognized on ESD ulcer.

EUS was performed to determine the invasion depth of the lesion prior to ESD. During EUS, a deaerated water-filling method was used for the observation using a 20 MHz miniature probe (Olympus Optical, Tokyo, Japan). The gastric wall was visualized as consisting of 5 layers. A hyperechoic line observed in the third layer was identified as the submucosa and a hypoechoic line observed in the fourth layer was identified as the muscularis propria. Lesions were classified into the following two groups according to the vascular structure identified by EUS in the third layer: group P, lesions with almost no hypoechoic areas likely to represent blood vessels or with ≤4 vessels of 50 *μ*m in diameter per field of view in the third layer (Figures 2 and 3); group R, lesions not classified as group P (Figures 4, 5, 6, and 7). In EUS, blood vessels were defined as structures visualized as hypoechoic, circular, or band-like areas in the third layer that could be recognized as vasculature. A structure was considered a ulcer scar when the third layer gradually disappeared or tapered off. During EUS, a single endoscopist (D. Kikuchi) classified each lesion into group P or R.

### 2.3. ESD

ESD was performed by skilled endoscopists who had experienced more than 100 cases of gastric ESD. Endoscopists under training were also allowed to perform the procedure under the guidance and supervision of the skilled endoscopists, who was blinded to EUS findings when performing ESD. ESD was performed using a GIF Q260J or 2TQ 260 M endoscope (Olympus Optical, Tokyo, Japan), a Flex or Dual knife (Olympus Optical, Tokyo, Japan) in all cases, plus a Hook knife (Olympus Optical, Tokyo, Japan) in some cases at the discretion of the operator, and an ICC 200 or VIO 300D high-frequency apparatus (ERBE, Tübingen, Germany). 

Some marks were placed around the lesion, Glyceol (Chugai Pharmaceutical, Tokyo, Japan) was injected locally, and an incision was made around the lesion. With the submucosa identified under direct view, the lesion was separated from the submucosa to obtain *en bloc* resection. Intraoperative bleeding was managed by coagulation hemostasis with the tip of the knife (swift coagulation, effect 4, 40 W) for mild bleeding or with hemostatic forceps (Pentax, Tokyo, Japan; soft coagulation, effect 4, 50 W) for moderate bleeding. For arterial bleeding or bleeding that could not be stopped with hemostatic forceps, clips (Olympus Optical, Tokyo, Japan) were used for hemostasis at the discretion of the operator. In cases of suspected injury or perforation of the muscle layer, a suture was made with clips at the discretion of the operator. On the day of ESD, the patient was fasted and given fluid replacement. On the day after ESD, abdominal, blood, and X-ray examinations were performed, and the operator decided when to resume eating based on the results of the examinations. One week after ESD, endoscopic examination was performed to confirm the absence of bleeding or exposed vessels. Patients who developed symptoms such as hematemesis or melena underwent urgent endoscopic examination, and those who required a hemostatic procedure for a post-ESD ulcer were considered to have developed postoperative bleeding.

### 2.4. Evaluation Items

This study was performed as a prospective observation study. The primary endpoint was the decrease in Hb after ESD compared with before ESD. The secondary endpoints were (1) total procedure time, (2) incidence of injury or perforation of the muscle layer during ESD, (3) incidence of clip use during ESD, (4) incidence of postoperative fever of ≥38°C, (5) incidence of postoperative bleeding, and (6) percentage of patients who resumed eating on the day after ESD. The total procedure time was defined as the interval from the start of marking to completion of resection. Perforation was defined as the presence of free air or mediastinal emphysema on postoperative X-ray. Muscle layer injury was defined as exposure and partial tearing of the muscularis propria, without perforation, during ESD.

### 2.5. Histological Evaluation

Pathological examination of the resected specimen was performed using parallel 2 mm thick sections stained with hematoxylin and eosin in accordance with the Japanese classification of gastric carcinoma [6].

### 2.6. Statistical Analysis

Data were analyzed using the unpaired *t*-test, chi-squared test, or Mann-Whitney's *U*-test as appropriate. A *P* value <0.05 was considered significant.

## 3. Results

*En bloc* resection was achieved by ESD in all cases. Of the 89 patients, 50 were classified into group P and 39 into group R. Patients background in each group is summarized in Table 1. No significant intergroup difference was observed in mean age, gender, macroscopic findings, mean maximum diameter of tumor, or mean maximum diameter of specimen. The proportion of patients with lesions in region L was significantly higher in group P while that of patients with lesions in region U was significantly higher in group R (*P* < 0.001). In terms of invasion depth, the proportion of patients with submucosal cancer was significantly higher in group R than in group P (*P* = 0.004).

The mean decrease in Hb, the primary endpoint, was 0.27 g/dL in group P and 0.35 g/dL in group R, with no significant difference between groups. Perforation occurred in only 1 (1.1%) patient in group R, and therefore no significant difference was found in the incidence of perforation between groups. The total procedure time was significantly longer in group R (105.4 min) compared with group P (65.5 min) (*P* = 0.002). The incidence of postoperative bleeding and postoperative fever was not significantly different between groups, whereas the incidence of muscle layer injury was significantly higher in group R (25.6%) than in group P (8.0%) (*P* = 0.02). The incidence of clip use was also significantly higher in group R (48.7%) than in group P (20.0%) (*P* = 0.004).

The incidence of muscle layer injury and that of clip use were not significantly different depending on the location of lesions, but tended to be higher in regions U/M and L in group R, respectively. The procedure time for lesions in region U/M was longer in group R, but not significantly (Table 3). The procedure time for lesions in region L was significantly longer in group R (120.5 min) than in group P (58.0 min) (Table 4). All group P patients with lesions in region L (37/37) resumed eating on the day after the operation; the proportion of such patients was significantly lower in group R (81.8%) (see Table 2).

## 4. Discussion

Invasion depth is an important criterion for selecting an appropriate treatment in early gastric cancer. Thus, prior to ESD, the invasion depth of a lesion is determined by various modalities, such as EUS. However, the diagnostic accuracy of EUS is controversial; some studies clearly demonstrate the usefulness of EUS for determining invasion depth in early gastric cancer [7] while others show the opposite [8]. When performing EUS to determine invasion depth, we sometimes see hypoechoic areas in the submucosa that are likely to represent blood vessels. Then, during ESD, we encounter blood vessels that appear to correspond to the hypoechoic areas on EUS. Based on these experiences, we retrospectively analyzed EUS images and demonstrated their predictive value for determining the risk of intraoperative bleeding and safety of ESD [5]. However, since the presence or absence of blood vessels may not be precisely determined by retrospective analysis, we decided to conduct the current prospective study to eliminate possible bias. This prospective study used a new definition of blood vessels identified by EUS. In the retrospective studies, lesions with ≥10 vascular structures per field of view or with vessels of ≥500 *μ*m in diameter were defined as “Rich” lesions and others as “Non-rich” lesions. In the present study, the definition of group P was changed to those with almost no hypoechoic areas likely to represent blood vessels in the submucosa or with 4 or fewer small vessels per field of view, and all other lesions not classified as group P were defined as group R. This change was based on our experience that higher reproducibility and credibility of diagnosis can be ensured by demonstrating the absence of blood vessels in the submucosa beneath the lesion.

The results of the present study revealed that the procedure time was significantly longer and that the incidence of clip use and muscle layer injury was significantly higher in group R than in group P, whereas no significant difference was seen in the decrease in Hb after ESD, the primary endpoint. A significant intergroup difference was observed in decrease in Hb in a previous retrospective study, but not in the present study. The most likely reason for this discrepancy is the operator's improved skill in performing ESD. In other words, in the previous study, the operator was less skillful, and bleeding was more likely to occur from vessel-rich lesions during ESD, while in the present study, improved skills in hemostasis and prophylactic hemostasis might have led to a decreased intraoperative bleeding volume. At the same time, this might have led to an increased time required for hemostatic procedures and subsequent significant increases in total procedure time and frequency of clip use. Another possible reason for the absence of significant difference in decrease in Hb after ESD is the major influence of factors other than intraoperative bleeding, such as patient physique and fluid replacement volume.

Given that ESD for lesions in region L has been associated with a lower volume of intraoperative bleeding than lesions in other regions [9], it is important to examine bleeding volume during ESD for lesions in each region. In the present study, each parameter was analyzed separately for regions L and U/M. Although no significant difference was observed for some endpoints due to small sample size, the procedure time tended to be longer and the incidence of muscle layer injury and clip use during ESD tended to be higher in group R than in group P, regardless of lesion location. In particular, the procedure time for lesions in region L was significantly longer in group R (120.5 min) than in group P (58.0 min). This finding indicates that ESD for lesions with many blood vessels in the submucosa by EUS is more likely to cause muscle layer injury and clip use, as well as requiring a longer procedure time. This is probably because for vessel-rich lesions, a longer time is required for hemostatic procedures, such as clip hemostasis, and because the incidence of complications, such as muscle layer injury, is increased due to reduced visibility of the surgical field by bleeding.

Ideally, ESD safety should be assessed on the basis of bleeding volume and incidence of perforation, and bleeding volume should be based on the actual amount of bleeding. However, since it is difficult to measure the actual amount of bleeding during ESD, the decrease in Hb was used as a surrogate measure for safety. In addition, the fact that perforation occurred in only 1 patient in group R demonstrates the safety of the procedure. Thus, other parameters, such as the incidence of muscle layer injury, incidence of clip use, and the proportion of patients who resumed eating on the day after ESD were also used as surrogate measures for safety. Overall, the incidence of muscle layer injury and clip use was significantly higher in group R than in group P. These parameters tended to be higher in group R regardless of the location of lesions, suggesting that the safety of ESD can be predicted by using EUS to some extent.

When performing ESD on lesions with abundant blood vessels in the submucosa, the operator should (1) carefully identify blood vessels under clear surgical field, (2) perform prophylactic hemostasis after identifying blood vessels, and (3) try to dissect the lesion at a deep layer of the submucosa [10]. In addition to these technical issues, EUS also provides important information for determining the treatment schedule and for selecting an appropriate operator. Specifically, when a lesion was found to be rich in blood vessels by EUS, a more skilled operator should perform the procedure in good time. For lesions expected to require a longer procedure time, the use of general anesthesia should also be considered.

There are several limitations to the present study. The first is that a single examiner evaluated EUS findings; multiple examiners may improve diagnostic objectivity and reliability. Second, the variability in skill levels of operators may also significantly affect the outcome of ESD [11]. We believe that the consistency of outcome was ensured in the present study, as ESD was performed by or under the supervision of a skilled endoscopist who had experienced more than 100 cases of gastric ESD. The third limitation is that frequency of submucosal cancer was significantly different. There is a possibility that ESD of submucosal cancer may be more difficult and danger than that of mucosal cancer. The last limitation is that we were not completely sure that the hypoechoic areas identified by EUS were truly blood vessels. It would have been ideal if the miniature probe had included a Doppler function. However, the miniature probe was our only option, as dedicated EUS systems with a Doppler function are unlikely to scan target lesions accurately because target lesions of this study were small. Development of more sophisticated instruments is another issue that needs to be addressed.

## 5. Conclusions

In conclusion, identifying blood vessels in the submucosa by EUS does not help in predicting the risk of worsening of anemia or occurrence of perforation, but may be helpful for predicting procedure time, risk of muscle layer injury, and use of clips. The preoperative use of EUS was effective for predicting the safety of ESD and procedure time and is thus considered useful for determining ESD treatment strategy.

## Figures and Tables

**Table 1:** Clinical characteristics of two groups.

	Group P	Group R	*P* value
Number of patients	50	39	
Gender (male/female)	37/13	33/6	0.23
Mean age (years ± SD^†^)	67.0 ± 8.9	66.2 ± 10.7	0.90
Location (U/M/L)	3/10/37	16/12/11	<0.001
Gross type (elevated/others)	15/35	13/26	0.74
Mean maximum diameter of tumor (mm ± SD)	20.2 ± 12.6	25.4 ± 19.7	0.18
Mean maximum diameter of specimen (mm ± SD)	41.0 ± 13.5	47.6 ± 19.2	0.06
Tumor depth (mucosal cancer/submucosal cancer)	47/3	28/11	0.004

^†^SD: standard deviation.

**Table 2:** Overall results of this study.

	Group P (*n* = 50)	Group R (*n* = 39)	*P* value
Mean decrease in Hb^†^ (g/dL ± SD^‡^)	0.26 ± 0.08	0.34 ± 0.10	0.56
Mean procedure time (min ± SD)	65.2 ± 39.9	105.4 ± 50.2	<0.001
Incidence of perforation, % (*n*)	0.0 (0)	2.6 (1)	0.25
Incidence of clip use, % (*n*)	20.0 (10)	48.7 (19)	0.004
Incidence of muscle injury, % (*n*)	8.0 (4)	25.6 (10)	0.023
Incidence of postoperative fever, % (*n*)	4.0 (2)	7.7 (3)	0.45
Incidence of postoperative bleeding, % (*n*)	4.0 (2)	7.7 (3)	0.45
Percentage of patients who resumed eating on the day after ESD, % (*n*)	84.0 (42)	82.1 (32)	0.81

^†^Hb: hemoglobin. ^‡^SD: standard deviation.

**Table 3:** Results for lesions in region U/M.

	Group P (*n* = 13)	Group R (*n* = 28)	*P* value
Mean decrease in Hb^†^ (g/dL ± SD^‡^)	0.21 ± 0.66	0.37 ± 0.67	0.55
Mean procedure time (min ± SD)	87.5 ± 51.3	99.5 ± 46.1	0.28
Incidence of perforation, % (*n*)	0.0 (0)	3.6 (1)	0.49
Incidence of clip use, % (*n*)	38.5 (5)	57.1 (16)	0.27
Incidence of muscle injury, % (*n*)	15.4 (2)	28.6 (8)	0.36
Incidence of postoperative fever, % (*n*)	7.7 (1)	10.7 (3)	0.76
Incidence of postoperative bleeding, % (*n*)	0.0 (0)	3.6 (1)	0.49
Percentage of patients who resumed eating on the day after ESD, % (*n*)	76.9 (10)	82.1 (23)	0.81

^†^Hb: hemoglobin. ^‡^SD: standard deviation.

**Table 4:** Results for lesions in region L.

	Group P (*n* = 37)	Group R (*n* = 11)	*P* value
Mean decrease in Hb^†^ (g/dL ± SD^‡^)	0.28 ± 0.57	0.27 ± 0.43	0.56
Mean procedure time (min ± SD)	58.0 ± 32.7	120.5 ± 58.8	0.002
Incidence of perforation, % (*n*)	0.0 (0)	0.0 (0)	
Incidence of clip use, % (*n*)	13.5 (5)	27.3 (3)	0.28
Incidence of muscle injury, % (*n*)	5.4 (2)	18.2 (2)	0.18
Incidence of postoperative fever, % (*n*)	2.7 (1)	0.0 (0)	0.58
Incidence of postoperative bleeding, % (*n*)	5.4 (2)	18.2 (2)	0.18
Percentage of patients who resumed eating on the day after ESD, % (*n*)	100 (37)	81.8 (9)	0.008

^†^Hb: hemoglobin. ^‡^SD: standard deviation.
